# Health Disparities Score Composite of Youth and Parent Dyads from an Obesity Prevention Intervention: iCook 4-H

**DOI:** 10.3390/healthcare6020051

**Published:** 2018-05-22

**Authors:** Melissa D. Olfert, Makenzie L. Barr, Rebecca L. Hagedorn, Lisa Franzen-Castle, Sarah E. Colby, Kendra K. Kattelmann, Adrienne A. White

**Affiliations:** 1 Davis College of Agriculture, Natural Resources & Design, Division of Animal and Nutritional Sciences, West Virginia University, G016 Agricultural Science Building, Morgantown, WV 26506, USA; mbarr6@mix.wvu.edu (M.L.B.); rlhagedorn@mix.wvu.edu (R.L.H.); 2 Nutrition and Health Sciences Department, University of Nebraska-Lincoln, 110 Ruth Leverton Hall, Lincoln, NE 68583-0806, USA; lfranzen2@unl.edu; 3 Department of Nutrition, University of Tennessee, 1215 W. Cumberland Avenue, 229 Jessie Harris Building, Knoxville, TN 37996-1920, USA; scolby1@utk.edu; 4 Department of Health and Nutritional Sciences, South Dakota State University, Box 2275A, SWG 425, Brookings, SD 57007, USA; Kendra.Kattelmann@sdstate.edu; 5 School of Food and Agriculture, University of Maine, 5735 Hitchner Hall, Orono, ME 04469, USA; awhite@maine.edu

**Keywords:** behavior, health disparities, nutrition, physical activity, family mealtime

## Abstract

iCook 4-H is a lifestyle intervention to improve diet, physical activity and mealtime behavior. Control and treatment dyads (adult primary meal preparer and a 9–10-year-old youth) completed surveys at baseline and 4, 12, and 24 months. A Health Disparity (HD) score composite was developed utilizing a series of 12 questions (maximum score = 12 with a higher score indicating a more severe health disparity). Questions came from the USDA short form U.S. Household Food Security Survey (5), participation in food assistance programs (1), food behavior (2), level of adult education completed (1), marital status (1), and race (1 adult and 1 child). There were 228 dyads (control *n* = 77; treatment *n* = 151) enrolled in the iCook 4-H study. Baseline HD scores were 3.00 ± 2.56 among control dyads and 2.97 ± 2.91 among treatment dyads, *p* = 0.6632. There was a significant decline in the HD score of the treatment group from baseline to 12 months (*p* = 0.0047) and baseline to 24 months (*p* = 0.0354). A treatment by 12-month time interaction was found (baseline mean 2.97 ± 2.91 vs. 12-month mean 1.78 ± 2.31; *p* = 0.0406). This study shows that behavioral change interventions for youth and adults can help improve factors that impact health equity; although, further research is needed to validate this HD score as a measure of health disparities across time.

## 1. Introduction

While there are many variations in how “Health Disparities” is defined [1,2,3], Braveman et al. (2014) defined health disparities as “worse health among socially disadvantaged individuals, specifically those of disadvantaged racial/ethnic groups” [4,5]. Although a clear classification system to identify individuals with health disparities has not yet been established, the idea that health disparities need to be eliminated, or at least mitigated, is widely accepted both nationally and internationally [6]. Health disparities adversely affect groups of people who have systematically experienced greater social and/or economic obstacles to being healthy, based on their racial or ethnic group, religion, socioeconomic status, gender, age, mental health (cognitive, sensory, or physical disability), sexual orientation or gender identity, geographic location, and other characteristics historically linked to discrimination or exclusion [1].

The United States (U.S.) Government has committed itself to the elimination of health disparities [1]. Every ten years, the U.S. Department of Health and Human Services publishes national objectives for improving the health of all Americans through health promotion and disease prevention [1,7]. Specifically, in Healthy People 2020, reducing health disparities was a primary objective with goals to “achieve health equity, eliminate disparities and improve the health of all groups”. The Centers for Population Health and Health Disparities (CPHHD) Program, through the National Institutes of Health (NIH), has called for a new direction in health disparity research, with renewed focus on addressing disparities at the individual and community levels, by utilizing interventions that improve health behavior choices [8].

Efforts to reduce and prevent health disparities may be especially important with youth populations because health at a young age sets the stage for health across the lifespan [9]. Schreier et al. examined health disparities as they impact adolescents [10]. They identified that adolescents growing up in a low socioeconomic status (SES), or “health disparate” environments, were more likely to experience negative health issues that could potentially continue into adulthood [10]. One such health issue disproportionally faced by youth living in “health disparate” environments, is obesity [11]. Obesity is linked to other co-morbidities such as type 2 diabetes, metabolic syndrome and some cancers [12]. Obesity in youth is strongly associated with increased risk of obesity and co-morbidities in adulthood [13,14,15].

In summary, health disparate populations tend to have poorer health outcomes and a deficiency of access to resources. However, what is lacking, is a mechanism to assess and identify which individuals are living in “health disparate” situations, through demographic, food security and food behaviors. Additionally, it is unknown if interventions can potentially improve or attenuate health behavior declines (through knowledge and access to health information) and whether they can ameliorate health disparities.

The hypothesis of this subproject was to test if participation in a childhood obesity prevention intervention, based on youth-adult dyads cooking, eating and playing together, would impact health disparities by using a “Health Disparate” (HD) score composite that was developed from the intervention’s program assessments. To test the hypothesis, the control and treatment participant groups’ HD scores were compared before and 4, 12 and 24 months into the childhood obesity prevention intervention.

## 2. Materials and Methods

iCook 4-H was a five-state (Maine, Nebraska, South Dakota, Tennessee and West Virginia) collaborative, childhood obesity prevention program that started in September of 2013. Researchers utilized a dyad approach of pairing a 9–10-year-old child and their adult primary food preparer. The iCook 4-H curriculum was developed by researchers and Extension professionals to allow youth and adults to learn-by-doing (experiential learning model) and capitalize on the social cognitive theory for behavior change [16]. Each state enrolled control and treatment dyads, with treatment dyads completing six, 2-h face-to-face iCook 4-H group sessions. The bi-weekly sessions focused on “cooking, eating and playing together.” Sessions were composed of culinary skill development and recipe preparation, a physical activity component, family mealtime and communication, and ended with goal setting. More detail on the specific iCook 4-H sessions is described elsewhere [17]. Newsletters and booster sessions were used for reinforcement over the two years. Upon completion of the program, researchers aimed to improve culinary skills, mealtime experiences and goal setting, as well as decrease sedentary time within these treatment dyads. Specifically, for the subproject described in this article, researchers sought to determine whether participation in a childhood obesity prevention intervention, iCook 4-H, could reduce participant’s health disparity burden by using a Health Disparity (HD) Score.

Ethical approval was obtained by each university’s Institutional Review Board prior to recruitment and the study was conducted in accordance with the Declaration of Helsinki. All subjects gave their written informed consent for inclusion before they participated in the study. This study was retrospectively registered (#54135351) on the ISRCTN online registry (www.isrctn.com).

iCook 4-H participants consisted of dyads that included a 9–10-year-old youth partnered with their primary adult meal preparer. Adult meal preparers had to be at least 19 years of age and prepare a majority of the meals in the household for the youth participant. Inclusion criteria required children to not be an age of 11 years old before 31 December 2013; have access to a computer with internet; and to be free from life-threatening illness or medical conditions, food allergies or activity-related medical restrictions that could prevent participation in a nutrition and physical activity-related intervention. Each state recruited participants from their local communities through Extension leaders, word of mouth, school mailings, and/or fliers. The data utilized will be a subproject of the main iCook 4-H intervention study.

Demographic characteristics (including gender, marital status, education level, and race/ethnicity) were collected via the baseline survey. Outcome measures were collected via survey from both control and treatment groups at baseline, 4 months, 12 months and 24 months. Outcomes from the surveys were used to develop a composite HD Score. The HD score was generated from a series of 12 questions including the USDA short form U.S. Household Food Security Survey (USHFSS) (5), participation in food assistance programs (e.g., Women, Infants and Children (WIC) or Supplemental Nutrition Assistance Program (SNAP) (1), food behavior (2), level of adult education completed (1), marital status (1), and race/ethnicity (1 adult and 1 youth question)). The USHFSS questions included HH3, HH4, AD1, AD2 and AD3, as described in the USDA survey module [18]. AD1a was excluded from the screener as not all participants were required to answer this question, as it was dependent on the response to AD1. Food behavior questions about food consumption patterns, were selected from the Expanded Food and Nutrition Education Program (EFNEP) Behavior Checklist [19]. Responses on the HD score were coded as binary variables of 0 or 1. The maximum score possible was 12 with higher scores representing increased health disparity for individuals.

All analyses were conducted using SAS (SAS^®^, Version 9.3) and JMP (JMP^®^, Version Pro 11) (SAS Institute Inc., Cary, North, CA, USA). Baseline demographic data are reported in frequencies and percentages. The difference between the control and treatment group’s baseline HD scores was analyzed using an independent *t*-test. To fit both random and fixed effects within the data, as well as the flexibility and individual variances and covariances, a Linear Mixed Model (LMM) was used. The best fitting model was determined through Akaike Information Criterion (AIC) and other goodness-of-fit indices. Time was treated as a continuous variable in the final model. Model fit of covariance structure was determined by Model Likelihood Ratio chi-squared test (LRT). The best fitting model for HD score included only a repeated statement of time, using an autoregressive (AR1) covariance structure, correcting for the degrees of freedom using the Kenward-Roger method and Restricted Maximum Likelihood (REML) estimation, with participant ID as the subject repeated variable. Significant effects were followed up with a post-hoc Tukey test for multiple comparisons.

## 3. Results

### 3.1. Participant Characteristics

There were 228 dyads that consented and enrolled into the iCook 4-H program from all five states with 195 that continued after the first session, with mean age of youth (9.35 ± 0.67 years) and adults (38.96 ± 8.04 years), as either control dyads (*n* = 77) or treatment (*n* = 151). Table 1 shows an additional demographic breakdown. Treatment youth were primarily White (67%) and female (52%). Treatment adults were also primarily White (70%) and female (83%), with just 64% being married and about 30% with a bachelor’s degree. Control youth were primarily White (61%) and male (61.0%). Control adults were also primarily White (65%) and female (81%), with about 64% being married and approximately 36% had completed some college. Based on chi-squared analysis, the only demographically significant differences were that more adults were female in the treatment group (*p* = 0.019). Over the 24 months there were 102 dropouts. Dropouts were more likely to be non-white (*p* = 0.03), not married (*p* = 0.001), have no post high school degree (*p* = 0.002), and use government food assistance programs (*p* = 0.005).

### 3.2. Pre- and Post-Intervention Health Disparity Score Composite

Complete data from 195 dyads were used at baseline to calculate control (*n* = 67) and treatment (*n* = 128) HD scores, with those missing any HD score variables removed. Baseline HD scores between groups were not statistically different (3.00 ± 2.56 control vs. 2.97 ± 2.91 treatment; *p* = 0.6632). In contrast, significant declines were found among the treatment from baseline to 12 months (*p* = 0.0047) and again from baseline to 24 months (*p* = 0.0354) (Table 2).

In Table 3 are the results of Linear Mixed Modeling (LMM) of HD scores across group (control and treatment) and time (baseline, 4, 12 and 24 months). HD score null model LRT was significant: (X^2^(1) = 496.12, *p* < 0.0001). The type 3 test for fixed effects of group interaction was not significant: F(1221) = 0.78, *p* = 0.3774, whereas the type 3 test for time interaction was significant: F(3394) = 3.67, *p* = 0.0124, and approaching significance for the type 3 group by time interaction: F(3394) = 2.58, *p* = 0.0533. After the Tukey post-hoc comparison test, significance was found in a treatment of 12-month time interaction (baseline mean = 2.97 ± 2.91, 12-month mean = 1.78 ± 2.31, *p* = 0.0406).

## 4. Discussion

Researchers found that after a 6-session childhood obesity prevention program, treatment dyads’ HD score was reduced significantly from baseline, at 12 and 24 months (Table 2). Upon further analyses, it was found that a significant interaction occurred between the treatment group and the 12-month time point for reduction in HD score after the intervention (Table 3). Compared to the treatment group, the control group had no significant decrease in their HD score (Table 2).

Although no definition has been universally agreed upon for Health Disparities [1,2,4,5], this study provided a formative look into health disparities by utilizing a series of demographic, food security and behavior questions, to capture and describe an individual’s level of health disparity into one composite score. Various measures were used to determine health disparities among this population; however, many previous researchers have used epidemiological approaches to define and capture health disparity changes [2,20,21]. However, to our knowledge, this is the first study to capture a composite score that encompasses numerous factors in an intervention setting. Braveman et al. (2006) stated that “Public health surveillance is certainly not sufficient to reduce health disparities”, showing a need for interventions such as iCook 4-H. Through this preliminary work, there is evidence for the importance of lifestyle interventions to decrease health disparities.

The utilization of a childhood obesity prevention program designed with a focus on behavior change for individuals and communities, aligns with the proposed recommendations of CPHHD [8]. It appears that CPHHD’s vision and recommendations for improving health disparities in populations through a community-based childhood obesity curriculum, can be successful. In the future, researchers should examine whether this intervention approach can be successfully utilized with diverse populations to address health disparities [22,23].

Although a broad range of health disparity factors were included in the composite score, some limitations of the current study include the validity and comprehensiveness of the tool. This current analysis provided a pilot analysis of the score and further testing and content validity needs to be conducted. It is acknowledged that this tool is largely focused on demographics and food security characteristics. Demographic variables such as marital status and race/ethnicity would not change as a result of an intervention. Future work to include knowledge and perceptions of health disparities in populations is warranted and validates this score as a measure of health disparities. For future analysis, a larger sample size and the use of cognitive interviews should be included to ensure that the components used in the score are representative of factors that determine health disparity. Future research projects designed to examine the effect of these interventions on health disparities should include a focus on rural communities. Although health disparities exist across the U.S., these disparities are pronounced in rural areas [22].

## 5. Conclusions

Overall, the dyads in the treatment group in this study had a decrease in their health disparities score after being provided with a lifestyle intervention focused on families cooking, eating and playing together. Although reducing health disparities was not the main outcome of the iCook 4-H intervention, this subproject aimed to provide an initial insight and understanding to developing a health disparities score. Health disparities may potentially be reduced after providing health information; thus, specifically understanding the perceptions of health disparities in a health intervention cohort is warranted for targeting interventions to improve these scores [24].

## Figures and Tables

**Table 1:** Baseline demographics of children and adults enrolled in the iCook 4-H program.

Demographics	Treatment		Control		*p*-Value
	*N*	%	*N*	%	
Total Population					
Dyads	151	66.2	77	33.8	
Child gender					
Male	73	48.3	30	61.0	0.1782
Female	78	51.7	47	39.0	
Child race					
White	96	63.6	47	61.0	0.5237
Black	16	10.6	9	11.7	
Hispanic	19	12.6	11	14.3	
Native American	5	3.3	1	1.3	
Asian	0	0	2	2.6	
Other	3	2.0	2	2.6	
NA	12	7.9	5	6.5	
Adult gender					
Male	9	6.0	12	15.6	0.0190 *
Female	126	83.4	62	80.5	
Adult Race					
White	106	70.2	50	64.9	0.1301
Black	13	8.6	5	6.5	
Hispanic	16	10.6	13	16.9	
Native American	3	2.0	0	0	
Asian	1	0.6	1	1.3	
Other	0	0	3	3.9	
NA	12	7.9	5	6.5	
Marital Status					
Married	97	64.2	50	64.9	0.5387
Single	17	11.3	9	11.7	
Divorced	11	7.3	10	13.0	
Committed	13	8.6	5	6.5	
Widowed	2	1.3	0	0	
NA	11	7.3	3	3.9	
Educational Level					
Elementary	7	4.6	2	2.6	0.1952
Some High School	1	0.6	2	2.6	
High School	22	14.6	5	6.5	
Some College	31	20.5	28	36.4	
Associates	20	13.2	8	10.4	
Bachelors	45	29.8	21	27.3	
Graduate	18	11.9	7	9.1	
Doctoral	5	3.3	2	2.6	
NA	2	1.3	2	2.6	

Demographic data represented in frequency and percentages. * *p* < 0.05, Chi-squared analysis.

**Table 2:** HD scores among groups across time.

Time	Control				Treatment			
	HD Score				HD Score			
	*N*	Mean	SD	*p*-Value	*N*	Mean	SD	*p*-Value
**Baseline**	67	3.00	2.56	---	128	2.97	2.91	---
**4 months**	51	3.04	3.04	0.9997	109	2.16	2.49	0.4265
**12 months**	48	2.75	2.91	1.0000	93	1.78	2.31	0.0047*
**24 months**	33	2.15	2.97	0.5742	81	1.41	2.14	0.0354*

Baseline was used as the reference group for each control and treatment comparison. HD scores across time within control and treatment groups reported in mean and standard deviation (SD). Tukey adjustment for group * time *p*-values.

**Table 3:** Mixed Regression Models for HD Score.

Variable	Category	Estimate	SE	*t*-Value	*p*-Value
**HD Score**					
Intercept		3.01	0.32	9.31	<0.0001 *
Group (referent: control):	Treatment	−0.10	0.40	−0.25	0.8047
Time	4 months	−0.97	0.19	−0.50	0.6190
	12 months	−0.04	0.26	−0.15	0.8845
	24 months	−0.63	0.34	−1.87	0.0624
Time*Role (referent: control; baseline):	Treatment * 4 months	−0.18	0.24	−0.78	0.4369
	Treatment * 12 months	0.66	0.32	−2.05	0.0406 *
	Treatment * 24 months	−0.09	0.41	−0.21	0.8299

Linear Mixed Model used to analyze the main effects of group and time on HD score. Significant effects were followed by multiple comparisons using Tukey adjustment. *p*-values for main effects and interactions are indicated. * *p* < 0.05.
